# Unmasking pipefish otolith using synchrotron-based scanning X-ray fluorescence

**DOI:** 10.1038/s41598-023-31798-z

**Published:** 2023-03-23

**Authors:** Vincent Haÿ, Sophie Berland, Kadda Medjoubi, Andrea Somogyi, Marion I. Mennesson, Philippe Keith, Clara Lord

**Affiliations:** 1 UMR 8067, Biologie Des Organismes Et Écosystèmes Aquatiques (BOREA), Sorbonne Université, Muséum National d’Histoire Naturelle, Université de Caen Normandie, Université Des Antilles, CNRS, IRD, CP26, 43 Rue Cuvier, 75005 Paris, France; 2 Synchrotron SOLEIL, 91192 Saint-Aubin, France

**Keywords:** Freshwater ecology, Ecology, Zoology

## Abstract

Scientists use otoliths to trace fish life history, especially fish migrations. Otoliths incorporate signatures of individual growth and environmental use. For many species, distinct increment patterns in the otolith are difficult to discern; thus, questions remain about crucial life history information. To unravel the history of such species, we use synchrotron-based scanning X-ray fluorescence. It allows the mapping of elements on the entire otolith at a high spatial resolution. It gives access to precise fish migration history by tagging landmark signature for environmental transition and it also characterises localised growth processes at a mineral level. Freshwater pipefish, which are of conservation concern, have otoliths that are small and fragile. Growth increments are impossible to identify and count; therefore, there is a major lack of knowledge about their life history. We confirm for the first time, by mapping strontium that the two tropical pipefish species studied are diadromous (transition freshwater/marine/freshwater). Mapping of other elements uncovered the existence of different migratory routes during the marine phase. Another major breakthrough is that we can chemically count growth increments solely based on sulphur signal as it is implicated in biomineralization processes. This novel method circumvents reader bias issues and enables age estimation even for otoliths with seemingly untraceable increments. The high spatial resolution elemental mapping methods push back limits of studies on life traits or stock characterisation.

## Introduction

Tropical island rivers are colonized by species with a life cycle adapted to these habitats–young, poor in nutriments, and subject to extreme climatic, hydrological and seasonal variation^1^. Most of the freshwater fauna is diadromous; organisms migrate between the ocean and freshwater^2^. In tropical environments, the main type of diadromy is amphidromy^2^. Amphidromous organisms, grow, feed and breed in the river. Eggs hatch in freshwater and the free embryos drift to the sea where they undergo a planktonic phase, before returning to the rivers to grow and reproduce^3^. In tropical rivers, the amphidromous life cycle has been confirmed for many fish species^4^. Among this freshwater ichthyofauna, twenty to thirty pipefish species (Syngnathidae) inhabit tropical rivers. The amphidromous life cycle is only suspected for freshwater pipefish^5,6^. The study of calcified structures (sclerochronology) gives access to the individual life history of fish. In teleosts, calcified structures show patterns linked to growth rate induced by environmental and endogenous factors^7^. Very few authors tried to analyse the otolith microstructure of Syngnathidae, and their efforts were poorly rewarded: observation of growth increments on otoliths of *Syngnathus biaculeatus* Bloch, 1785 was unsuccessful^8^ and during the attempt to study the life history of weedy sea dragons *Phyllopteryx taeniolatus* (Lacepède, 1804)*,* authors were unable to find the otoliths in the cranium^9^. Syngnathidae otoliths are highly challenging: excessively small (less than 400 µm long and 200 µm thick), fragile and without discernible growth increments. Therefore, the study of freshwater pipefish otoliths, both focusing on microstructure or microchemical analysis has never been undertaken.

The otolith, a paired structure found in the inner ear, provides key information on life history characteristics, such as age, growth rate, age at maturity, migratory behaviour and information about the environment an individual may have inhabited^10^. Their growth is continuous throughout the fish’s life; they are made of successive discrete layers of calcium carbonate (CaCO_3_), crystalline microstructural growth increments that are deposited over a nucleus on a protein matrix^11^. These increments are usually formed on a regular basis^11,12^ with alternating light zones (L-zones) (areas rich in calcium carbonate) and dark zones (D-zones) (rich in organic material). Counting the growth increments gives an estimate of the fish’s age or of the duration of specific events. As increments are continuously formed, they incorporate chemical elements withdrawn from the environment in which the fish evolved at the time of the crystallisation. Otoliths are useful tools to reconstruct the environmental and physiological past of teleosts and the information is revealed by chemical or structural microanalyses. However, extrinsic and intrinsic drivers, like salinity, temperature ontogeny or feeding/growth may affect biomineralization processes^13^, but certain trace elements (such as strontium (Sr), barium (Ba) or manganese (Mn)) have commonly been used as environmental tracers for fish^14^. Even though metabolic processes can affect the carbonate accretion of otoliths, trace elements are incorporated into the otolith and reflect their environmental availability^13,15^. For example, Sr content of otoliths is positively correlated to the Sr concentration of the surrounding water, which is high in seawater and low in freshwater^16^. Consequently, Sr:Ca ratio has been widely used to reconstruct diadromous fish migratory movements^17,18^. There is growing body of work on otolith microchemistry that provide insights into fish life history events due to the availability of advanced analytical tools.

Laser ablation inductively coupled plasma mass spectrometry (LA-ICP-MS) is an efficient method that has been used routinely in many studies on trace elements of otoliths on numerous taxa^19–21^ and even in the study of other calcified structures such as bones^22^. Despite the advantages (easily accessible, low cost and quick in terms of data acquisition) of this method, it shows three main issues^23^. Firstly, the line transect created by the laser beam commonly performed from the otolith core (nucleus) to the outer edge does not provide complete information on otolith chemistry. Secondly, the laser spot size is 20 to 30 µm: it is highly destructive and prevents any subsequent analysis and it is too large for the study of specific areas (e.g. the core) and for otoliths of small sizes. Thirdly, the accurate quantification of trace elements can be problematic, owing to the detection limits of this device. Researchers have explored non-destructive methods mapping trace element variation throughout otoliths. Synchrotron X-ray fluorescence (XRF) has seldom been used in otolith chemistry studies although it shows real promise in this field. Indeed, two-dimensional maps obtained by XRF produce complete images of the dynamics of elemental incorporation in the otolith^23^, thus allowing the characterisation of the elemental spatial heterogeneity more finely (down to 0.5 µm in this study), in particular in samples of small sizes. The pioneer use of synchrotron XRF methods on otoliths was by analysing Sr:Ca ratio to characterize eel migrations^24^. Synchrotron methods are used to track fish migration in polluted areas with selenium (Se), an element with weak ionization properties and therefore difficult to detect by LA-ICP-MS^25^. They also are applied to study archaeological and modern fish otoliths to identify the potential of these methods, especially to better distinguish biogenic signal from *post-mortem* and post-depositional sources of alteration^26^. Numerous applications are possible and the ability to detect multiple elements at low concentrations simultaneously and at a fine scale is necessary for studying otolith microchemistry. Although this method has many advantages, mapping in two-dimensions also has disadvantages. Acquisition time (complete mapping, and data processing) remains much higher that producing a transect^27^ and synchrotron study cost are still high and less accessible than LA-ICP-MS studies^28^.

We present in this paper an innovative method to reveal life history traits of two freshwater pipefish species, *Microphis nicoleae* (Haÿ et al., in press) distributed from Papua New Guinea to the Solomon Islands and *Microphis brachyurus* (Bleeker, 1854) distributed from Sri Lanka to French Polynesia by otoliths microchemical analysis. We used synchrotron-based scanning X-ray spectromicroscopy (an elemental imaging technique with analytical capabilities) and quantitative data mining to assess individual diadromous migration by mapping Sr level changes in the otolith at a global and hyperfine resolution. We explored the possibility of scoring otolith growth increments using a chemical element based counting method thanks to XRF approach. In addition to presenting a novel method in the study of otoliths, this work provides basic knowledge in the ecology and biology of freshwater pipefish; knowledge that is compulsory to implement conservation and management measures.

## Results

### Diadromous pipefish

**Figure 1:**
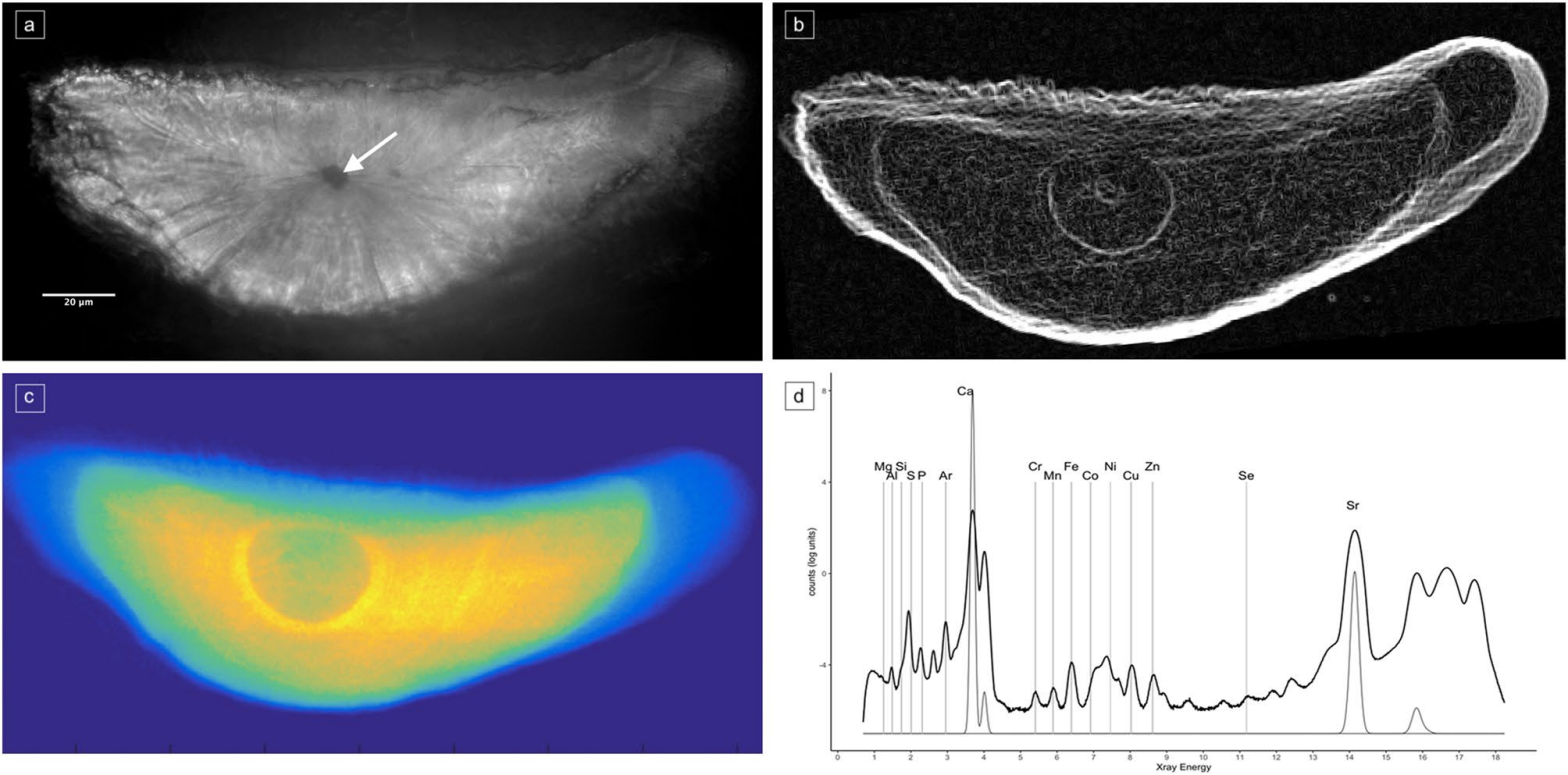
Application of synchrotron X-ray fluorescence (XRF) imaging and spectrometry to tag landmark signature for environmental transition in the otolith. (**a**) Polarized light microscopy of the otolith (*M. nicoleae* sagitta) ground to the nucleus (arrow). (**b**) Edge detection in raster image of the sum-XRF spectra unmasking zonation in the otolith. (**c**) Elemental map of strontium (Sr). (**d**) The elemental composition of otolith identified from the XRF sum-spectra. Both elemental identification and quantification are possible.

**Figure 2:**
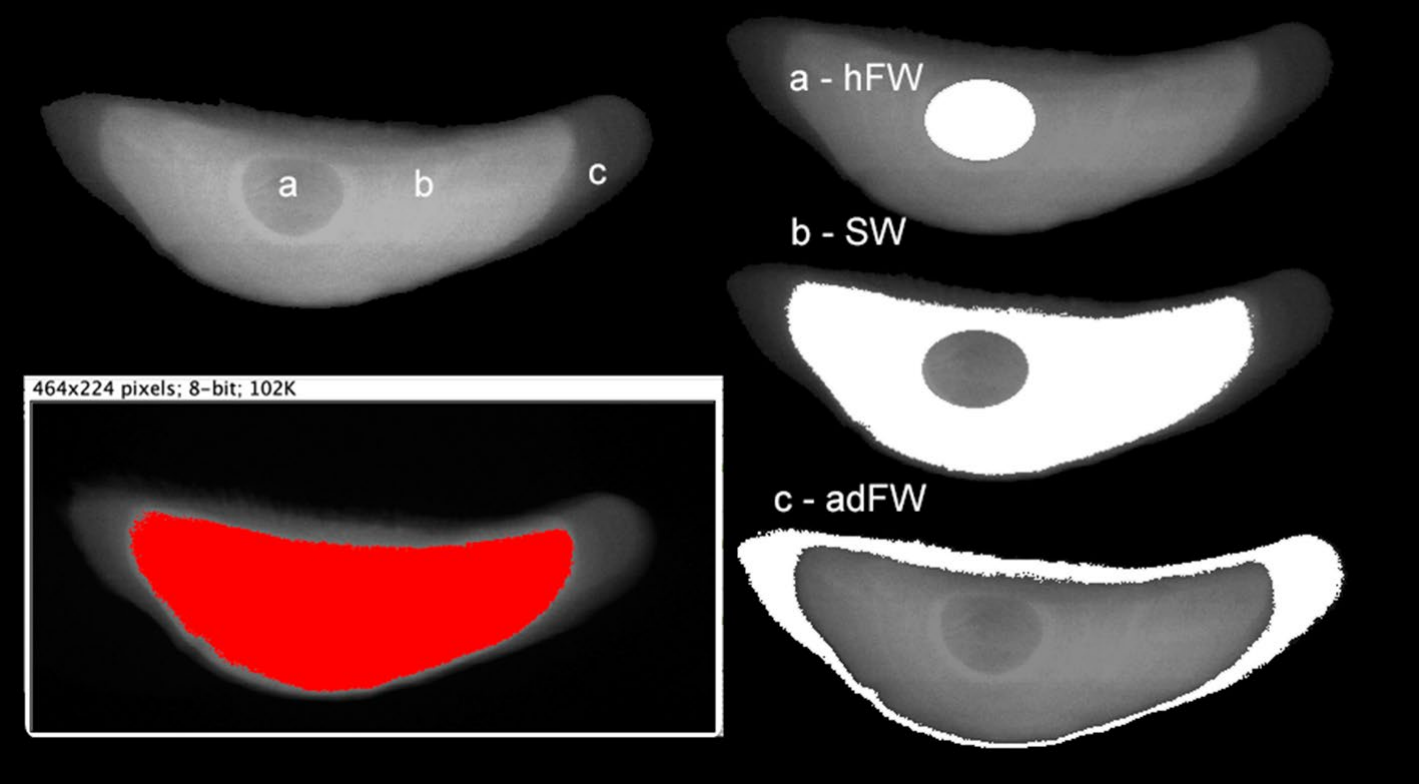
Sketch showing the design process for the delimitation of interest areas from the otolith samples. The specific masks were obtained by thresholding the XRF strontium (Sr) maps to fit in with the zonation exhibited in grey level scale (on the left). For each area a mask was created (right panel), with the central core (**a**), the high Sr content area (**b**) and the distal margin (**c**) respectively corresponding to *'hatching freshwater*' (hFW), '*sea water*' (SW) and '*adult freshwater*' (adFW) conditions. These masks served as boundaries for discrete quantitative analysis on each designed region of interest.

**Figure 3:**
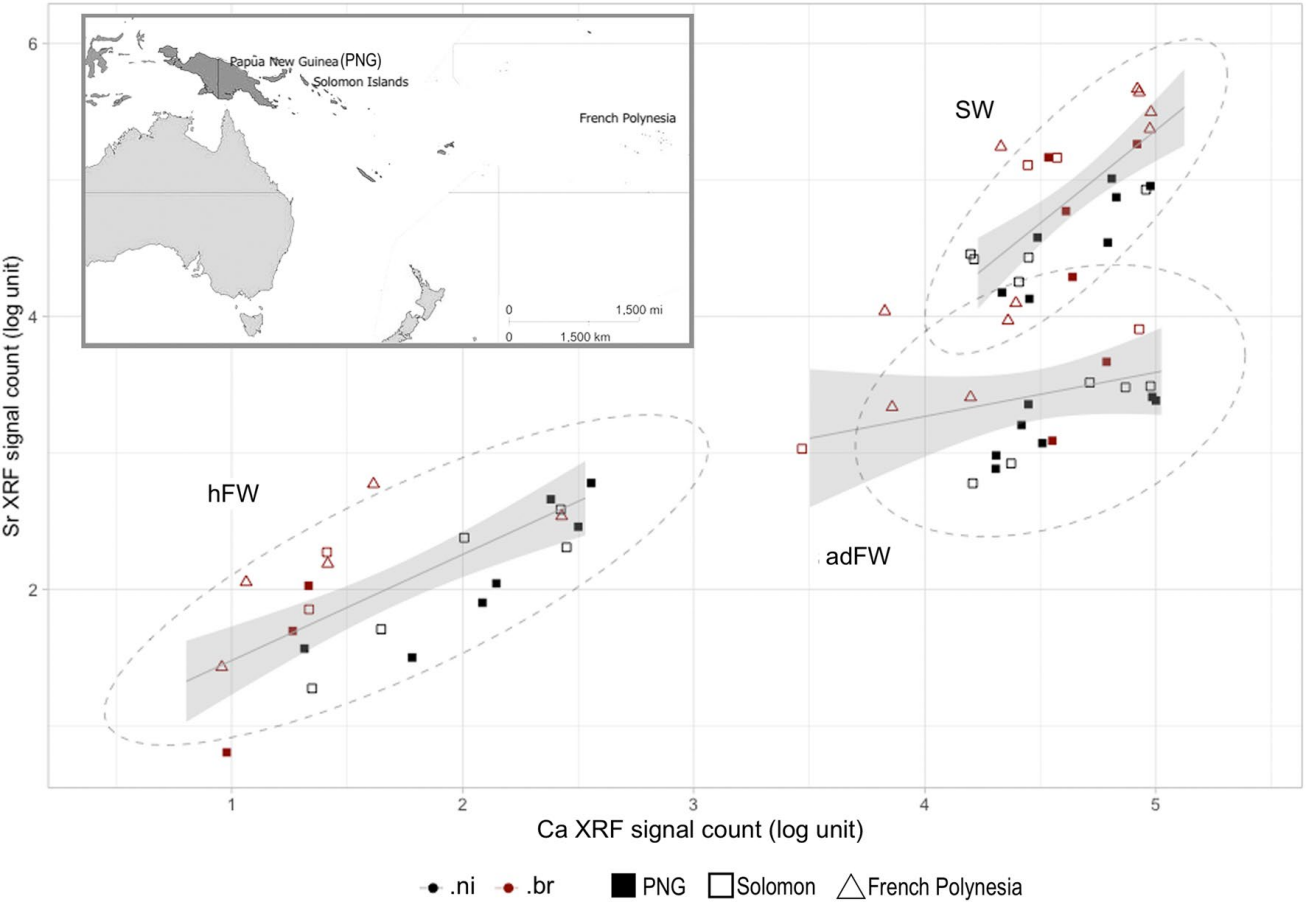
Distribution of strontium (Sr) as a function of calcium (Ca) element between each area (*hatching freshwater* (hFW), *sea water* (SW) and *adult freshwater* (adFW)) with a map location of the sampling (top inset). Besides, a continuum can be drawn from the early post hatching group (hFW) to the otolith edge group (adFW) which stands for sustained Sr-Ca relationship correlated with individual growth, a discrete cluster (SW) signing for environment transition step. (.ni): *Microphis nicoleae*; (.br): *Microphis brachyurus*; PNG: Papua New Guinea.

XRF images uncovered, for the first time, an amphidromous life cycle for two freshwater pipefish, *Microphis nicoleae* and *Microphis brachyurus* (Figs. 1c, 2 and 3). For all individuals (N = 19) from all localities (Papua New Guinea -PNG-, Solomon Islands and French Polynesia) and both species, Sr density shows the same patterns. Different Sr densities allow the delimitation of three concentric zones from the core to the edge, corresponding to different environments and, in the case of amphidromous species, to different development stages. Sr-depleted zone near the core, corresponds to a freshwater phase where larvae hatch, called *hatching freshwater* (hFW). Sr-enriched zone, corresponds to a juvenile seawater phase, called *sea water* (SW). Sr-depleted zone, near the edge corresponds to an adult freshwater phase, called *adult freshwater* (adFW) (Figs. 2 and 3). Otoliths observed under polarized light microscopy and SEM do not exhibit particular marks like a check mark. Check marks, indicating the recruitment of larvae from the ocean to estuaries, are commonly found in other amphidromous fishes^29^, but these fish usually undergo a metamorphosis during the recruitment. The check mark is more related to metamorphosis than to environment transition^30^.

### Trace element variations

**Figure 4:**
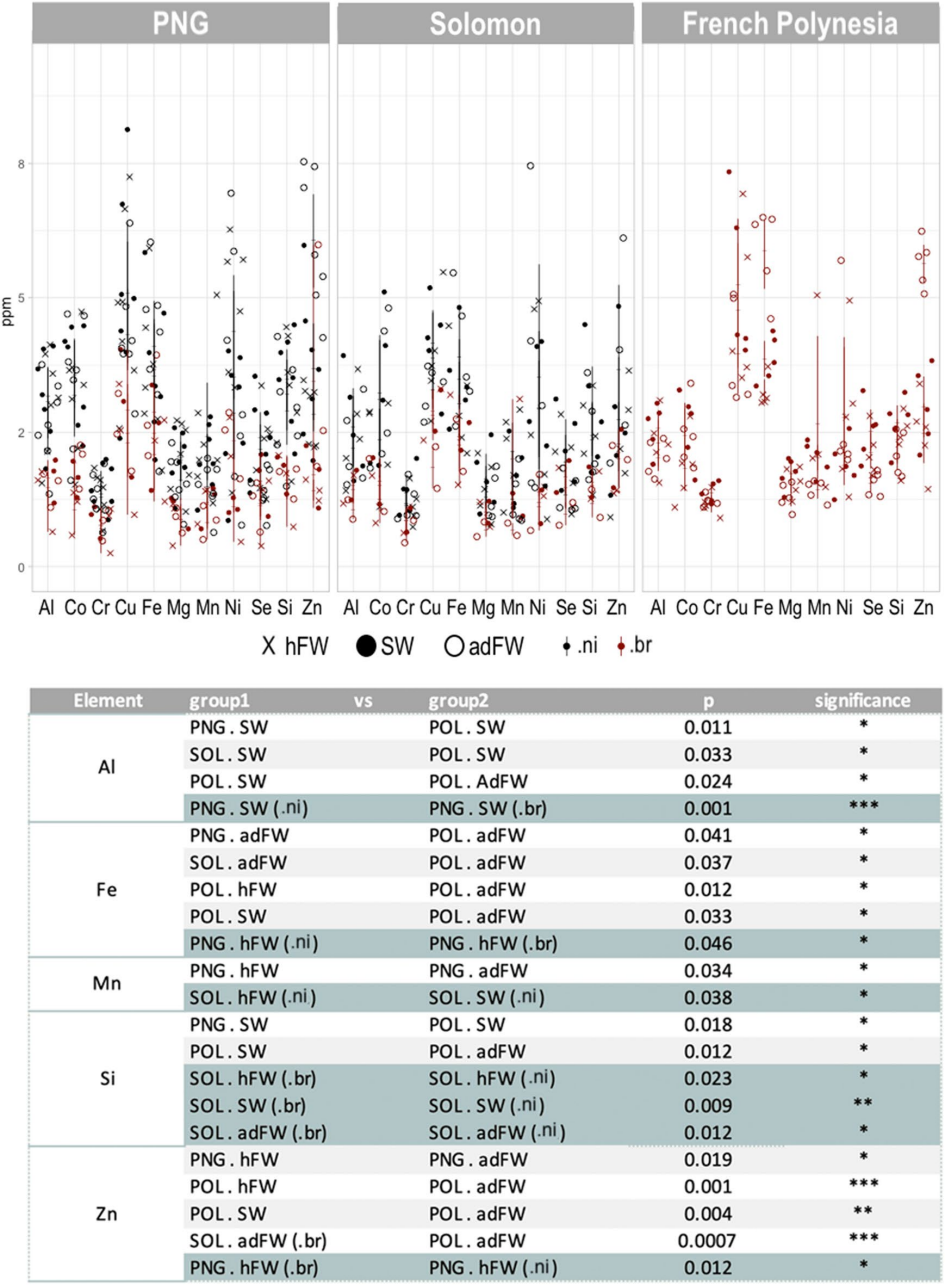
Environmental discrimination fingerprints in the otolith. Trace element (ppm) variations in the otolith zones corresponding to the *'hatching freshwater*', '*sea water*' and '*adult freshwater*' conditions (respectively hFW, SW and adFW) and split into the geographic region to which the samples belong. At the bottom are provided the corresponding statistics performed using non-parametric pairwise then post-hoc tests. Argon stability was checked across the samples to ensure robustness and reliability of the data (Supplementary Figure 1). (.ni): *Microphis nicoleae* ; (.br): *Microphis brachyurus*; PNG: Papua New Guinea.

Analysis of trace elements highlighted differences at three levels (Fig. 4): (i) between localities (*e.i.* French Polynesia, PNG and Solomon Islands); (ii) inside the otolith, between the different areas (*e.i.* hFW, SW and adFW); (iii) between species (*e.i. M. nicoleae* and *M. brachyurus*). Differences are specifically found in the SW area. Specific trace elements like aluminium (Al) and silicon (Si) separate individuals sampled in French Polynesia from those sampled in Solomon Islands and PNG (Fig. 4). We also observed for the same locality, different elemental compositions (for Al, magnesium (Mg), Mn, iron (Fe), Se, sulphur (S) and zinc (Zn)) according to the different areas of the otolith (hFW, SW and adFW). For example, significant differences appear between hFW/SW and adFW areas in French Polynesia for Fe and Zn (Fig. 4). Finally, some elements seem to have a different incorporation according to the species. Otolith composition in Al, Fe, Mn, Si and Zn vary between *M. nicoleae* and *M. brachyurus* from the same locality. For example, significant differences appear between the two species, notably in the Solomon Islands for Si (Fig. 4).

### Otolith increments

**Figure 5:**
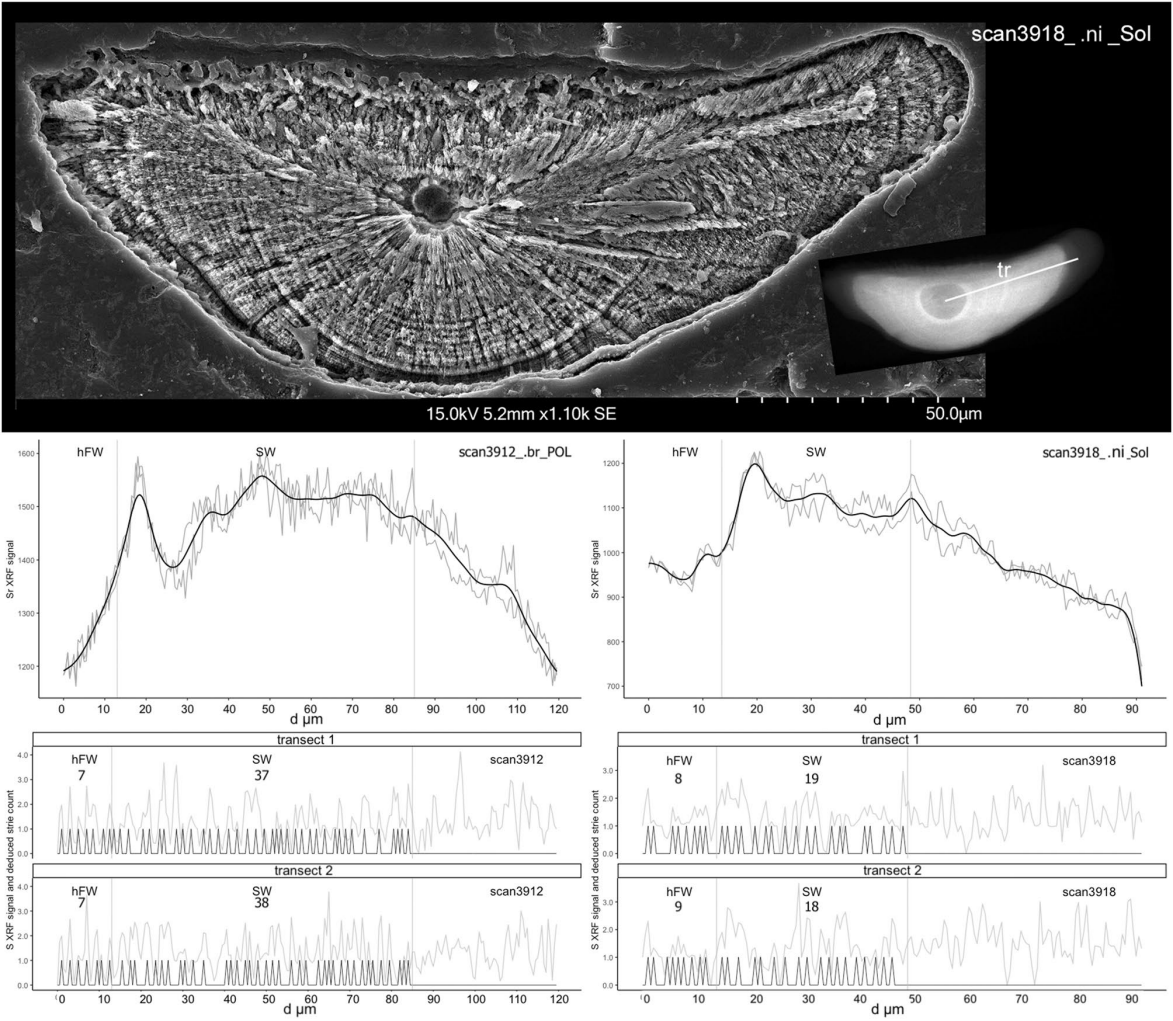
Interpolation of linear coordinates of strontium (Sr) and sulphur (S) signal allowed by XRF high spatial resolution mapping to uncover the growth increment checks in the otolith. On top of the figure, picture shows SEM image of otolith after the mild etching. Transects were performed on XRF scans for Sr and S starting from the nucleus to the edge (see inset). Middle graphs present boundaries between fresh- and seawater (hFW and SW respectively) which were deduced from the shifts after smoothing (black line) of Sr signal. Bottom graphs show S traces on the transects, with the environment transition limits deduced from Sr level major changes and the incremental growth exposed by deciphering the S sawtooth shaped trace rhythm.

**Figure 6:**
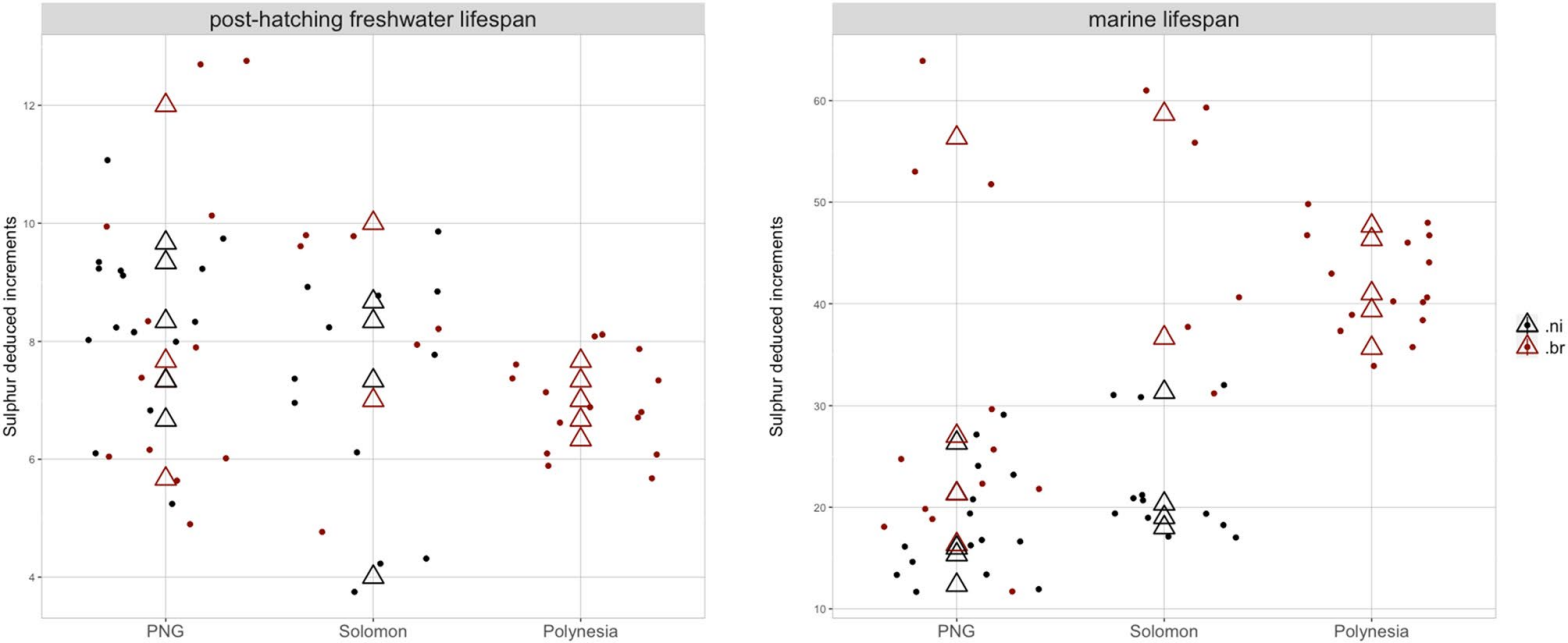
Plot summarizes the individual increment counting deduced from the sulphur (S) trace (three replicates in each sample) along their post hatching freshwater downstream migration (left) and their spreading time span within sea water environment (right) for each locality and species. (.ni): *Microphis nicoleae*; (.br): *Microphis brachyurus*.

S spectra analysis uncovered a dynamic of S incorporation in the otolith linked to the alternation of growth increments (Figs. 5 and 6). Based on the three areas delimited on each otolith after Sr mapping, we were able to estimate for each specimen the number of growth increments of the hFW phase (*i.e.* time laps between hatching in freshwater and downstream migration into the marine environment) and the SW phase, also called in many studies on amphidromous fishes, the pelagic larval duration (PLD), that is the time spent at sea before recruiting back to freshwater.

The mean number of increments of the hFW phase was estimated at 7.5 ± 1.5 for *M. nicoleae* and 7.7 ± 1.7 for *M. brachyurus* (no significant difference *W* = 41.5, *P* value = 0.5413). SW (or PLD) mean numbers of increments were estimated at 19.7 ± 5.8 for *M. nicoleae* and 38.6 ± 13.1 for *M. brachyurus* (PLD of *M. brachyurus* significantly longer than PLD of *M.* *nicoleae W* = 89.5, *P* value = 0.0026). For 4 otoliths and for the same transect, S-based increment counts were compared to the counts based on SEM picture. Results for manual *versus* “chemical” mathematical process respectively were 10/10; 12/12; 14/11; 14/12 (Supplementary Figure 2).

## Discussion

In this study, thanks to high resolution specificity of XRF mapping, we were able to detect fine scale and multi-level information from mute otoliths, such as Syngnathidae otoliths. Trace elements in the otolith can be used as natural tags and can trace fish habitat at a specific time in its life^31^, thus tracing migration patterns throughout a fish’s life. We observed a diadromous life cycle for the two freshwater species studied and probable discrimination of populations and species through trace element compositions in their otoliths. In addition, XRF analysis offers to disclose growth increments in the challenging otoliths by using S signal.

### Sr:Ca mapping and diadromous life cycle

The present study is the first investigation of the otolith microchemistry for two freshwater pipefish, *M. nicoleae* and *M. brachyurus.* Until now, an amphidromous life cycle was only suspected for freshwater pipefish^5,6^ and has never been studied precisely due to the complexity of otolith analysis in Syngnathidae. Their Small size (less than 400 µm), their fragility and the lack of discernible growth increments complicate the use of traditional microchemical analysis for these structures. Otolith analysis of these two species revealed alternating high and low Sr density from the nucleus to the edge (Figs. 1c and 2). This alternating density pattern of Sr in fish otoliths is considered as proof of migratory movements between marine and freshwater environments^17^ and can be considered as a proxy of Sr:Ca ratio. High Sr:Ca ratios in fish otoliths are widely accepted as proof of fish exposed to a marine environment^32,33^ such as during a marine larval stage^34^; as opposed to low Sr:Ca ratio, which reflects occupation of fish exposed to freshwater. Consequently, otolith microchemistry supports a diadromous cycle and more specifically an amphidromous cycle for the two species with two environmental transitions from freshwater to seawater to freshwater during the individual life. For many amphidromous species, the transition between seawater and freshwater during the recruitment can be assessed by the presence of distinctive marks on the otolith, such as a check mark^35^ but no such mark was detected on pipefish otoliths. The appearance of a mark (a check mark) in the increments of the otolith is due to the sudden energetic expenditure for morphological changes, thus leading to cessation of growth and tightening of increments. Some amphidromous fish species undergo drastic morphological, physiological and behavioural changes when they return to rivers^29,36^. These changes, defined as metamorphosis are under endocrine control, and especially thyroid hormones^37^. In freshwater pipefish, and in Syngnathidae in general, morphology between newly hatched larvae and juveniles in rivers is similar^6^, most likely implying a lack of metamorphosis, even if important physiological processes for osmoregulation must take place during the return to river for these species.

### Multi-level trace element variations

The three zones inside the otolith delineated by Sr transition reflect the different habitats encountered by the individual during its life. Complete otolith mapping allows the study of trace element variations at different levels: individual level (diadromous life cycle as discussed above), population level (differences in elemental composition between localities) and species level (differences in incorporation of some elements according to the species). This multilevel information retrieved by synchrotron analysis shows that the use of this innovative method brings otolith microchemical analysis to a whole new dimension compared to other methods traditionally used.

The differences between localities for Al, Fe, Mn, Si, and Zn (Fig. 4) could be explained by the geological heterogeneity of the Pacific area. Pacific Islands have various origins, such as volcanic chains (arc or hotspot) or continental fragments or atolls^38^. This leads to a variety of crystalline basements inducing specific elemental signatures that can be incorporated in otoliths^39^. Differences in trace elements appeared between PNG, the Solomon Islands and French Polynesia mostly for the marine pelagic phase (SW) (Fig. 4). These differences could be the result of different migratory routes taken during the marine phase. Many surface currents exist in the Indo-Pacific area which have a great influence on the juveniles during their marine dispersal and therefore on the population structure^40^. Further studies with additional samples (location and individuals) are needed to clarify the geochemical signature.

Differences between species may be due to intrinsic factors. Physiological processes (like growth and gonad development) can modify blood and therefore otolith composition^20^. Diet can also be responsible for some variations. *M. nicoleae* and *M. brachyurus* live in sympatry in the Solomon Islands and PNG; the difference in snout length between the two species (long for *M. brachyurus* and short for *M. nicoleae*) may cause changes in their diet, allowing the two species to share the same habitat while exploiting different trophic niches. Currently, the impact of feeding on elemental composition of otoliths is not well understood^13^.

### S-based counting of otolith increments

Otoliths show a series of growth structures that are formed on a regular basis^15^. These structures, called increments, grow according to time series and have been widely used for age estimation in many fish species^41^. Microstructural studies of the otolith are essential to characterize more precisely fish life history. Unfortunately for pipefish otoliths the increment count is hampered by a lack of resolution of standard microscopy methods. Syngnathidae microstructural analysis must rely on new methodologies and this study shows that otolith increments can be chemically counted. Indeed, the density of S in the otolith has a cycle nature along the growth axis (Figs. 5 and 6). We were able to confirm increment counts using S by comparing with counting carried out on standard SEM images which disclosed some areas available for counting. Authors have shown that S is correlated with D-zone (areas rich in organic material) and S is assumed to be incorporated in the otolith as a component of the organic matrix^42^. Higher S concentrations in D-zones are probably mostly associated with S-rich proteoglycans^43,44^. The role of sulphated macromolecules in biominerals reported in the outcome of organic matrix scaffolding and mineral nucleation^45^ supports the characterisation of otolith growth pattern by using S element trace. This S-based counting of increments is an innovative and reliable method for age determination in fish. Until now, age determination is based mainly on counting increments on the otolith surface^46^. However, age determination may vary due to otolith preparation and to the reader who can have different interpretations of the growth patterns and usually several readers are needed to enhance robustness process^21^. Performing the increment count using microchemical analysis (here with S) limits reader bias, making fish age estimation more accurate.

Previous studies validate daily accretion of growth increments^47,48^, on which we bring the assumption that the growth increments of the two species studied here were formed on a daily basis. The first increment near the core is supposed to be formed at hatching^50^. With this new method we have estimated the mean duration of the post-hatching phase for *M. nicoleae* (7.5 ± 1.5 days) and *M. brachyurus* (7.7 ± 1.7 days) but also, the mean SW migration duration for *M. nicoleae* (19.7 ± 5.8 days) and *M. brachyurus* (38.6 ± 13.1 days). Few studies have focused on the time spent in rivers for the newly hatched larvae just before drifting to the sea. Migration to the sea exhibits several risks, such as predation or starvation. Amphidromous species have found ways to circumvent this issue: a downstream migration at sea^51^, a large number of eggs^52^, an early larval hatch^53^, a timing of hatching to coincide with elevated river flows^54^ or a positive phototaxis for newly hatched larvae allowing passage in river flows^55^. For example, *Sicyopterus lagocephalus* (Gobiidae) larvae die after seven days if they have not reached the sea^56^ and pro-larvae of *S. lagocephalus* need to downstream to the sea in a maximum of 96 h to pursue their development^57^. Mean time spent in river after hatching for the two pipefish species studied here seems to be longer than expected for amphidromous fish. We hypothesize that this greater time spent in the river may also be an efficient strategy for these species. Indeed, it might allow juveniles to complete part of their development and therefore increase their survival during the marine phase. However, the crucial lack of knowledge complicates the study of their biology and life history.

These mean marine lifespan durations seem to be shorter than for other amphidromous fish for which duration of three to six months is most frequently observed^29^. The difference observed in our study between the two species in their marine duration could be explained by their geographical distribution. Indeed, *M. brachyurus* has a more widespread distribution in the Indo-Pacific than *M. nicoleae*. *M. brachyurus* is known from Madagascar, Indonesia, Japan to French Polynesia while *M. nicoleae* is known from Papua New Guinea and the Solomon Islands. The dispersal potential and the range size of a species are positively correlated in the Indo-Pacific^58^, driven by the spatial distribution of habitat and dispersal barriers. The longer marine phase would suggest greater dispersal capacities and allows to colonize distant geographical islands, hence the widespread distribution range of *M. brachyurus* rather than the range of *M. nicoleae*. But PLD is not always correlated to the species range^30,48^; other biological and environmental factors are involved in the dispersal process^59^. Yet, very few studies have focused on Syngnathidae otoliths; thus, the reliability of the daily increments and age estimation which is still controversial^60,61^ needs to be validated in freshwater pipefish in order to confirm age estimation for these species. Indeed, in the case of diadromous fish, estimation of the time spent in each environment (freshwater and ocean) bring key information for these species on their migratory capabilities^48^.

## Conclusion

The study of freshwater pipefish otoliths represented a complex challenge, with little hope of retrieving any information on the life history of these highly valuable species. However, we have shown in this work that the use of synchrotron XRF nano-imaging is particularly powerful and opens new possibilities in otolith analysis, both in the study of elemental composition and the microstructure. A major asset is the non-destructive aspect of the method, offering the possibility to undertake subsequent analyses. Overall, we retrieved information on three different levels: (i) the validation of the amphidromous life cycle of the two freshwater pipefish studied here, *Microphis brachyurus* and *Microphis* *nicoleae*; (ii) the differences in the composition of trace elements shows the existence of probable different populations for each species and/or the use of different migratory routes during the marine phase of the life cycle; (iii) the differences found between species probably reflect different behaviours (feeding, reproduction etc.) of species sharing the same localities, opening the possibility of future works on microhabitat preferences. In addition to this multilevel analysis (individual, population and specific), the complete mapping of otoliths allowed the study of some elements present in minute quantities (ranging from 1.02 ± 0.4 ppm in Solomon location individuals Cr in adFW area, to 5.9 ± 0.8 ppm in French Polynesia location individuals Fe in adFW area). Moreover, temporal patterns were uncovered for some of them such as S. The periodical variation of the concentration of sulphur could be linked to the production of the protein rich increments formed on a daily basis: the count of S peaks is a new and powerful method for individual age estimation.

This study greatly improves our knowledge of freshwater pipefish as their otoliths were previously impossible to study with other traditionally used methods. Our results obtained on these two species pave the way for much future work, for instance tackling the nucleus, maternally inherited and formed in the very early developmental stages, but due to its size is yet an even higher challenge and for which synchrotron XRF analysis may allow to push the limits. This work will be undertaken shortly on freshwater Syngnathidae to deepen our knowledge on the migrating behaviour of these species. Other perspectives include to pursue unravelling habitat transitions use as key life trait in the sustainability of species in fragmented environments especially when subjected to daunting threats. Regardless of species, broader knowledge is needed, such as genetic or morphological studies for the implementation of conservation measures and for habitat protection.

## Material and methods

### Samples

The present study was performed on dead animals, which have been sampled during several past field missions and preserved in 95% ethanol, suitable storage medium for fish to be used in otolith microchemistry^62^, in the National Museum of Natural History of Paris collection. Live animals were not handled. Ten specimens of *M. nicoleae* were collected from the Solomon Islands (N = 4) and Papua New Guinea (N = 6) and nine specimens of *M. brachyurus* were collected from the Solomon Islands (N = 2), Papua New Guinea (N = 3) and French Polynesia (N = 4). All fish were collected in freshwater. The standard length was measured using a dial caliper (Mitutoyo) (Table 1).

**Table 1:** Sampling location and description.

Collection Number	Id	Scan	Species	Standard length (mm)	Snout length (mm)	Sex	Otolith	River	Sample location
MNHN-IC-2023–0045	19055SD	Scan3917	*Microphis nicoleae*	82.26	3.86	M	sagitta	Walindi-1	Papua New Guinea
MNHN-IC-2021–0338	17693SG	Scan3922	*Microphis nicoleae*	93.66	3.99	M	sagitta	Hoskin_Road	
MNHN-IC-2023–0446	19185LG	Scan3923	*Microphis nicoleae*	87.98	3.57	M	lapillus	Gavuver	
MNHN-IC-2023–0446	19179SD	Scan3929	*Microphis nicoleae*	84.66	3.51	M	sagitta	Gavuver	
MNHN-IC-2023–0446	19183SG	Scan3939	*Microphis nicoleae*	69.41	3.41	F	sagitta	Gavuver	
MNHN-IC-2023–0446	19176SD	Scan3940	*Microphis nicoleae*	90.86	3.99	M	sagitta	Gavuver	
MNHN-IC-2023–0446	19185SG	Scan3941	*Microphis nicoleae*	87.98	3.57	M	sagitta	Gavuver	
MNHN-IC-2021–0318	17763SD	Scan3927	*Microphis brachyurus*	154.23	21.74	M	sagitta	Swamp_Rangihi	
MNHN-IC-2021–0318	17765SD	Scan3928	*Microphis brachyurus*	129.38	18.64	M	sagitta	Swamp_Rangihi	
MNHN-IC-2021–0318	17766SG	Scan3943	*Microphis brachyurus*	122.49	17.49	F	sagitta	Swamp_Rangihi	
MNHN-IC-2021–0336	14962SD	Scan3918	*Microphis nicoleae*	64.37	3.33	F	sagitta	Rannungga	Solomon Islands
MNHN-IC-2021–0336	14961SG	Scan3919	*Microphis nicoleae*	83.33	4.21	F	sagitta	Rannungga	
MNHN-IC-2023–0047	18253SD	Scan3920	*Microphis nicoleae*	97.65	4.04	M	sagitta	Isabel_Rakata	
MNHN-IC-2023–0047	18253LD	Scan3921	*Microphis nicoleae*	97.65	4.04	M	lapillus	Isabel_Rakata	
MNHN-IC-2023–0048	18268SG	Scan3924	*Microphis nicoleae*	103.9	4.61	M	sagitta	Isabel_Rakata-2	
MNHN-IC-2021–0331	19190SD	Scan3916	*Microphis brachyurus*	101.98	14.99	F	sagitta	Vage	
MNHN-IC-2023–0049	18256LD	Scan3926	*Microphis brachyurus*	151.1	21.1	M	lapillus	Isabel_Kolopikassa	
MNHN-IC-2023–0050	PFV52SD	Scan3912	*Microphis brachyurus*	125.32	17.04	F	sagitta	Papenoo	French Polynesia
MNHN-IC-2023–0051	PFV06SD	Scan3913	*Microphis brachyurus*	105.02	13.01	F	sagitta	Papenoo	
MNHN-IC-2023–0052	PFV39LD	Scan3914	*Microphis brachyurus*	109.36	14.29	M	lapillus	Papenoo	
MNHN-IC-2023–0052	PFV39SD	Scan3915	*Microphis brachyurus*	109.36	14.29	M	sagitta	Papenoo	
MNHN-IC-2023–0053	PFV42SG	Scan3933	*Microphis brachyurus*	104.15	14.07	F	sagitta	Papenoo	

### Otolith preparation

Sagittal otoliths were extracted from the fish under a binocular magnifier (Leica SE9), rinsed in MilliQ water and kept dry in Eppendorf tubes until they were individually embedded in epoxy resin (Araldite 2020, Escil, France). Each otolith was ground on a frontal section down to the core, obtaining the greatest length with a reduced loss of material, using carbide silicon 800-grain abrasive discs to expose the core then a finer 4000-grain disc (Escil, France). The embedded otoliths were polished with diamond paste (Escil, France) and rinsed and sonicated in MilliQ water. The final preparation is less than 300 µm thick.

### Synchrotron-based scanning X-ray microscopy

The X-ray fluorescence (XRF) spectromicroscopy was performed on the full-size area of the otolith section surface (Fig. 1a) at the scanning spectro-microscopy nano-imaging station at the Nanoscopium beamline of the Synchrotron Soleil (Saint-Aubin, France). The incident X-ray beam of 15 keV energy was focused by a Kirckpatrick-Baez mirror to a size of 0.3 × 0.3 μm^2^. This device allows a fine microscopic definition at pixel size of each beam spot on which spectroscopy is then performed. The distribution maps were collected under continuous scanning (FLYSCAN) mode by two Si-drift detectors for elements from atomic number 13 (Al) to 38 (Sr) principal emission lines (K and L)^63^*.* All the data used in this study was obtained with 0.5 µm pixel size and 40 ms acquisition time. Each sample total scanning dataset was gathered as Hierarchical Data Format version 5 (HDF5) to be processed for multiple element quantification.

Detailed elemental distribution maps of the main components, Ca and Sr, together with trace element abundances have been obtained thanks to the high spatial resolution of synchrotron XRF nano-imaging. From this, Ca and Sr standalone distribution maps allowed to define the sample boundaries and the within limits of the specific areas of interest used in this study.

### Delimitation of specific areas

Masking method was used to design limited areas of interest prior to data integration. The XRF images for Sr and Ca elements were processed using Fiji software, open access Java-based image processing program^64^. The distribution maps were digitalized and subjected to thresholding then disentangled on purpose using basic boolean operators applied to the images to draw the three concentric regions of interest, that were converted after digitalization into masks: the central core, the high strontium density rim, and the distal low strontium density border. The masks were then applied to the HDF5 files by the image calculator tool of Fiji software to treat the specific areas of interest and to process the related data subsets. To do so, the sum XRF-spectrum corresponding to the z-axis profile of each stack was extracted from the HDF5 images and then was fitted by PyMca software^65^. The overall mask designing process is sketched in Fig. 2.

### Quantitative data mining

Quantification of elements above the detection limits was obtained using the PyMca software without the need to recur to standard reference materials thanks to in-house computational models developed to apply matrix effects correction for absorption and fluorescence of the X-rays interaction processes in the sample. Hence X-ray signal intensity is highly correlated to the analyze concentration at pixel size (0.5 µm) scale.

The intensities (counts per dwell time) of the XRF peaks of the identified element from the XRF spectra were computed from the HDF5 files. Quantitative elemental abundances (ppm) were then extracted after appropriate calibration and were reported to the surface of the depicted area (in pixels). Apart from the otolith major components Ca and Sr, the method allowed quantification of a set of trace elements: Al, cobalt (Co), chromium (Cr), copper (Cu), Fe, Mg, Mn, nickel (Ni), Se, Si and Zn (plus S as ingrowth tag) (Supplementary Table 1). The set of detectable peaks over noise offered by the system sensitivity is given (sample full map) in Fig. 1.d.

### Statistical analysis

For descriptive linear correlation and statistical analyses, the collaborative open source R project was used (R Development Core team 2010). The general patterns of Ca and Sr correlation throughout the areas of the otolith were displayed using ggplot2 package, geom_smooth command line and ‘lm’ as the method.

Differences in trace element features for the different sample locations and related to each specific area within the otolith were subjected to Kruskal–Wallis non-parametric rank test to determine whether there were significant differences among the groups (each group represents the interaction between sample location and specific otolith area). This method was consistent with regards to the data format, Normal distribution is not required (the limited sampling does not allow robust Normal distribution test) but the assumption of independent observations was met. Results were then analyzed using the Tukey's Honest Significant Difference test (Tukey HSD post-hoc) as a non-parametric rank test to determine from all pairwise comparison whether the differences among the distribution from different locations and within the otolith areas were statistically significant. This was done since no assumption of independent observations nor equal variances was met by using pairwise_*t*_test() fonction from rstatix package, with "two.sided", var.equal = FALSE and pool.sd = FALSE as options. Null hypothesis of equal mean ranks was considered and the low* p* value allowed rejecting the null hypothesis.

### Growth modeling using biomarkers from XRF acquisition data

Minute changes in the distribution of Sr and S input throughout the otolith were searched for at the finest spatial resolution allowed by the applied synchrotron beam (0.5 µm). Sr is meant to sign for changes in salinity^16^ and S is a key element of life as a protein constituent (S amino acid)^42^; in the context of otoliths, S can be used as organic matrix protein fingerprint.

In each otolith sample, edge detection image of all merged XRF signals unmasking zonation in the otolith (see Fig. 1b), Sr and S XRF scan data were loaded as images using Fiji software. Ground zero-point coordinates were determined from the core center in the edge detection image. Both Sr and S scan images were stacked so that they can be manipulated equally. Sr and S deposit continuums were recorded as quantitative data along a transect going from ground zero-point coordinates (deemed the otolith origin) to the most distal edge of the otolith. Using the image stacks, the variation of each element over the same transect was exported as z-profile data.

Both graphs were searched for the turnpoints. This was done using the collaborative R project and the pastecs package ‘turnpoints’ function that determines the number and the position of extrema (turning points) after data transformation into a time series. As for the Sr signal course over the transect, the turnpoint search was dedicated to indicate the spots where the curve suffered extreme slope steepness. The curve smoothing was performed by running ‘locpoly’ function of package ‘KernSmooth’ to fit a regular course by means of piecewise linear interpolation prior to the search of the turnpoint position within steep changes flanks.

The raw S trace was subjected to the turnpoint search with the method described above; the position of each S peak was collected. The position of the turnpoints deduced from the Sr data along the transect used to mark the environmental transitions from fresh to seawater condition (and vice-versa) were placed equally on the S data to frame the environmental transition boundaries. Then the number of turnpoints and the position within this frame allowed calculating the otolith increment rate for each area of interest separately.

### Scanning electronic microscopy preparation

After mapping trace elements with XRF, cuts of embedded otoliths were prepared for scanning electronic microscopy (SEM). Otoliths were etched with 1.25% solution of ethylene diamine tetra-acetic acid (EDTA) for 3′30’’. After etching, otoliths were mounted on the SEM stub and coated with a 5 nm thick layer of platinum under Leica EM ACE600. Otoliths were scanned by MEB Hitachi SU3500 at a voltage of 15 keV. Increments present on the otoliths were counted. Results of age estimation were compared to the S-based increment calculation method described above over the same transects for the 4 otoliths.

## Supplementary Information

Supplementary Information 1.

Supplementary Information 2.

Supplementary Information 3.

## Data Availability

All data generated or analyzed in this manuscript are included in the supplementary files.
