# Supplementary material for: Unmasking pipefish otolith using synchrotron-based scanning X-ray fluorescence
Source: Sci Rep. 2023 Mar 23;13:4794. doi: 10.1038/s41598-023-31798-z (PMC10036667; doi:10.1038/s41598-023-31798-z)
Supplement: Supplementary file 1 — Supplementary Information 1. [file 41598_2023_31798_MOESM1_ESM.pdf]

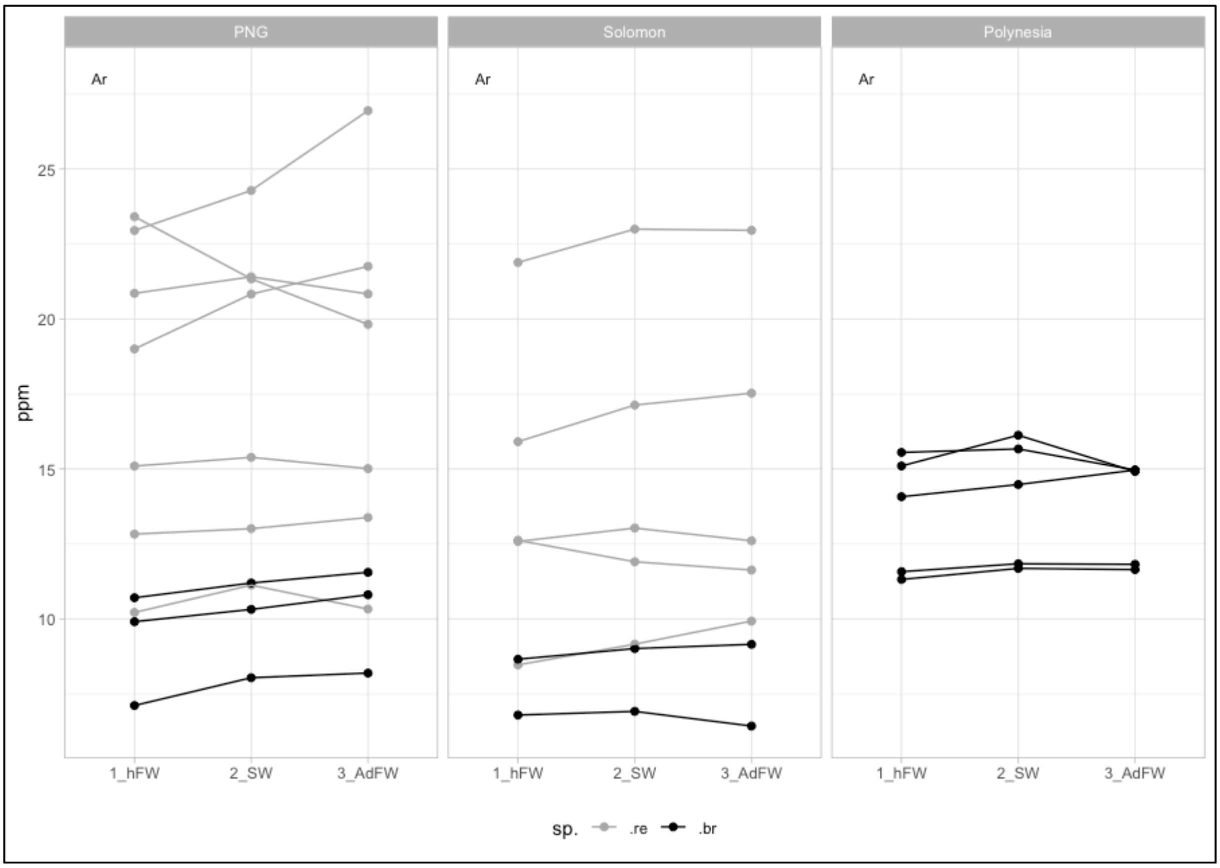

Supplementary Figure 1: Argon measurements (ppm) for each sample, covering each region of interest of the otolith (hatching freshwater (hFW), sea water (SW), adult freshwater (adFW)). The graph shows argon (Ar) stability over every sample, which meets the requirements to ensure robustness of minor element quantification.

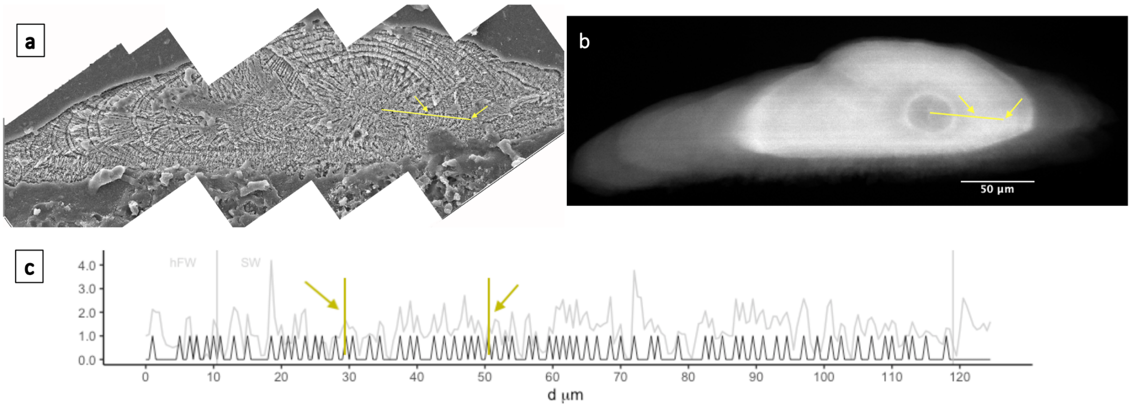

sple	increment count by reading MEB image	increment count using sulphur trace process	
Scan3928_17765SD	12	12	present fig.a-b-c
Scan3939_19183SG	10	10	data not shown
Scan3918_14962SD	14	11	data not shown
Scan3915_PFV39SD	14	12	data not shown

Supplementary Figure 2: Validation of otolith increment counts using sulphur (S) trace process. Control region where increments were available for reading on scanning image of the otolith after mild etching were subjected both to eye-reading and S-based increment calculation method. The picture shows the landmark position of the control region on the MEB image (a) and the XRF scan (b). The S trace and the deduced increment suite is plotted on graph (c). Region for cross check counting is located in-between the arrows. The table (bottom of the figure) shows the results for each age estimation method.
